# 3D Printing of Collagen/Oligomeric Proanthocyanidin/Oxidized Hyaluronic Acid Composite Scaffolds for Articular Cartilage Repair

**DOI:** 10.3390/polym13183123

**Published:** 2021-09-16

**Authors:** Chung-Fei Lee, Yung-Heng Hsu, Yu-Chien Lin, Thu-Trang Nguyen, Hsiang-Wen Chen, Sasza Chyntara Nabilla, Shao-Yi Hou, Feng-Cheng Chang, Ren-Jei Chung

**Affiliations:** 1 Department of Chemical Engineering and Biotechnology, National Taipei University of Technology (Taipei Tech.), Taipei 10608, Taiwan; calvin.lee7770@gmail.com (C.-F.L.); thutrang03121996@gmail.com (T.-T.N.); jessica840220@gmail.com (H.-W.C.); f10955@ntut.edu.tw (S.-Y.H.); 2 Bone and Joint Research Center, Chang Gung Memorial Hospital, Linko 33305, Taiwan; hsuyh@cgmh.org.tw; 3 Department of Orthopaedic Surgery, Chang Gung Memorial Hospital, Linko 33305, Taiwan; 4 College of Medicine, Chang Gung University, Taoyuan 33302, Taiwan; 5 Department of Materials, Imperial College London, London SW7 2BP, UK; yu-chien.lin17@imperial.ac.uk; 6 Department of Materials, University of Oxford, Oxford OX1 3PH, UK; sasza.nabilla@oriel.ox.ac.uk; 7 School of Forestry and Resource Conservation, National Taiwan University, Taipei 10617, Taiwan; fcchang@ntu.edu.tw

**Keywords:** articular cartilage, porous scaffolds, 3D printing, collagen, oligomeric proanthocyanidin, oxidized hyaluronic acid

## Abstract

Articular cartilage defects affect millions of people worldwide, including children, adolescents, and adults. Progressive wear and tear of articular cartilage can lead to progressive tissue loss, further exposing the bony ends and leaving them unprotected, which may ultimately cause osteoarthritis (degenerative joint disease). Unlike other self-repairing tissues, cartilage has a low regenerative capacity; once injured, the cartilage is much more difficult to heal. Consequently, developing methods to repair this defect remains a challenge in clinical practice. In recent years, tissue engineering applications have employed the use of three-dimensional (3D) porous scaffolds for growing cells to regenerate damaged cartilage. However, these scaffolds are mainly chemically synthesized polymers or are crosslinked using organic solvents. Utilizing 3D printing technologies to prepare biodegradable natural composite scaffolds could replace chemically synthesized polymers with more natural polymers or low-toxicity crosslinkers. In this study, collagen/oligomeric proanthocyanidin/oxidized hyaluronic acid composite scaffolds showing high biocompatibility and excellent mechanical properties were prepared. The compressive strengths of the scaffolds were between 0.25–0.55 MPa. Cell viability of the 3D scaffolds reached up to 90%, which indicates that they are favorable surfaces for the deposition of apatite. An in vivo test was performed using the Sprague Dawley (SD) rat skull model. Histological images revealed signs of angiogenesis and new bone formation. Therefore, 3D collagen-based scaffolds can be used as potential candidates for articular cartilage repair.

## 1. Introduction

The largest joint in the human body is the knee, which carries the body’s weight and is majorly responsible for our smooth mobility and stability; especially, it is used heavily in sports. Common defects in load-bearing are the main reason for the easily weakened and damaged knee cartilage. Accumulation of gradual wear and tear, repetitive actions, or a sudden injury can cause lesions in cartilage, potentially leading to early post-traumatic degeneration, chronic repetitive micro-trauma, and developmental defects well-known as osteochondritis dissecans (OCD) [1,2]. Millions of people, worldwide, are affected by cartilage defects annually. In the United States, an estimated 200,000–300,000 patients have undergone cartilage surgery [3].

Articular cartilage is a highly specialized connective tissue of diarthrodial joints that has a low cell number and is non-neural, lymphatic, and avascular. This type of cartilage consists of hyaline cartilage; once damaged, it takes much longer to heal due to a lack of blood vessels and nerve supply; moreover, chondrocytes cannot freely migrate to the site of injury from an intact health site [4,5]. In such cases, reparative processes are not available for repair of the tissue; therefore, articular cartilage has a limited capacity for self-regeneration after injury.

To date, options for articular cartilage treatment can be categorized into injectables and surgical treatments. The injection treatment often uses drugs such as analgesics, steroids [6], or hyaluronic acid [7], as well as the injection of platelet-rich plasma [8] to provide temporary pain relief. If damage to the cartilage is severe enough, surgical treatment is required to restore the structural integrity of the joint cartilage [9]. The microfracture surgery technique, autologous chondrocyte transplantation surgery, and matrix-induced autologous chondrocyte implantation (MACI) allow cells to grow on a collagen matrix to restore the biological tissue; however, in spite of repair work being performed, the structural and functional properties of cartilage are not fully restored [10]. Consequently, the repair of articular cartilage defects is still one of the greatest challenges, as it is also costly [11].

Three-dimensional printing is an ideal technology to be used in tissue engineering to produce scaffolds for the repair or replacement of damaged tissues and organs [12]. In this technique, scaffolds with controllable biodegradability, adjustable pore structures, and excellent biomechanical properties are fabricated. Biodegradability is an important factor to be considered in the design of scaffolds, as it allows cells to produce their extracellular matrices to repair damaged areas [13]. The porous structure of scaffolds also plays an important role in improving the efficiency of biomaterials. It is beneficial for cell growth, which helps promote the diffusion and metabolism of cells. Kohane et al. reported that a total porosity of more than 90% is suitable for osteoblast growth and for promoting cell attachment, growth, and differentiation [14]. Further, mimicking characteristic mechanical properties to fuse with cartilage architecture at a macroscopic level is vital for scaffolds during the implantation stage and to eventually sustain these properties for the regeneration of new tissue [15].

In this study, we used a wide range of applications of microextrusion bioprinting to fabricate collagen type I-based scaffolds. Collagen is a biodegradable and bioabsorbable material. Likewise, repeating motifs formed by the alpha chain of hydroxyproline-proline-glycine [16] generate excellent biocompatibility and biosafety properties, and show low antigenicity. According to Chevallay et al., with its extremely low immune response, rich porous structure, and high permeability, collagen can regulate cell morphology, adhesion, migration, differentiation, and other functions [17,18].

Collagen type I accounts for 20–30% of the protein in the body, and is widely distributed in the intercellular matrix and the extracellular matrix (ECM) of vertebrate connective tissue, alongside hyaluronic acid (HA). In recent years, many studies have proved that HA exhibits good hydrophilicity, biodegradability, permeability, plasticity, biocompatibility [19], low immunogenicity, and viscoelasticity, and can affect cell proliferation [20], migration [21], and differentiation [22]. Transparent HA is used for the treatment of joint diseases. A high concentration of hyaluronic acid as a column of lubricating fluid into joints may reduce pain in the affected area [23,24,25]. Furthermore, HA may play a role of acting as a crosslinker with other compounds. In this study, sodium periodate was used to oxidize the hydroxyl groups on HA to open the ring and generate an active aldehyde group; this oxidized hyaluronic acid (OHA) acts as a cross-linking agent via a network formation with the amine group of collagen.

Although HA is available for cross-linking to collagen, this compound still possesses low mechanical properties. Oligomeric proanthocyanidins (OPCs) are polyphenolic antioxidant cross-linkers [26] and flavonoid-condensed tannins [27]. Similar to tannins, OPCs can easily form hydroxylated structures, and cross-link with carbohydrates and proteins to form insoluble complexes [28]. Flavonoids have also been studied to stimulate the production of osteoblasts and inhibit their effects [29]. The application of OPCs to produce biomedical materials for the repair of biological tissues [30] by using them as crosslinkers to cross-link collage prolongs the degradation time of collagen, and supports tissue regeneration. Compared with cells that commonly use the agent glutaraldehyde, OPCs are 120 times less toxic [31]. In orthopedics, whether in skull reconstruction [32] or bone repair [33], scaffolds containing OPCs promote new bone formation. In another dentistry-related study, an OPC stabilized the structure and strength of adhesion of dentin collagen through the cross-linking of collagen with dentin, thereby increasing its mechanical properties [34].

Therefore, the aim of our study was to perform studies related to the repair of articular cartilage using three-dimensional scaffolds composed of natural materials such as collagen (C), oxidized hyaluronic acid (OHA), and oligomeric proanthocyanidins (OPCs) as biocompatible cross-linkers. Bone marrow mesenchymal stem cells of rats were used for in vitro experiments to evaluate cell cytotoxicity, cell viability, and cell proliferation. In vivo studies were performed on skulls of Sprague Dawley (SD) rats to observe the formation of new bone.

## 2. Materials and Methods

### 2.1. Materials

Collagen (Coreleader Biotech Co., Ltd., New Taipei City, Taiwan), OHA with a molecule weight of 700~1100 KDa (Coreleader Biotech Co., Ltd., New Taipei City, Taiwan), and OPCs (Compson Trading Co., Ltd., Taichung, Taiwan) were used to prepare the biological inks. Acetic acid, sodium periodate, and ethylene glycol were obtained from Sigma-Aldrich Inc. (St. Louis, MO, USA). Deionized water (DI H_2_O) and phosphate-buffered solution (PBS) were used as the main solvents in all the experiments.

### 2.2. Fabrication of Scaffolds

A collagen solution (40 mg/mL) was produced by mixing 1600 mg of collagen fibers with 40 mL of a 0.5 M acetic acid aqueous solution. Next, the mixture was stirred for 3 d at 4 °C. Oxidized hyaluronic acid (OHA) was prepared using 8000 mg of hyaluronic acid (HA) dissolved in 800 mL DI water. Sodium periodate (NaIO_4_) (240 mg) was added to the hyaluronic acid solution and stirred for 24 h at room temperature until completely dissolved. Ethylene glycol (16 mL) was then added, and the mixture was stirred for 30 min to stop the reaction. The solution was dialyzed with DI water for 3 days (dialysis bag MWCO = 3.5 K). Thereafter, the solution was refrigerated at −80 °C for 48 h and then dried completely using a freeze dryer. OHA was dissolved in DI water to prepare 1 mg/mL, 5 mg/mL, and 10 mg/mL solutions. A 10 mg/mL OPC solution was prepared by mixing 300 mg of water-soluble OPC powder in 30 mL of DI water.

Collagen/oligomeric proanthocyanidin/oxidized hyaluronic acid composite scaffolds were prepared using a Cellink Inkredible 3D Bioprinter. Collagen (40 mg/mL) was printed using a 3D printer employing a 22G blue metal needle and a pressure of 120 kPa. Initially, the Z-axis was set to 12.1 cm. Four layers of collagen were printed to create a cylindrical-shaped scaffold with an 8 mm diameter and 1.2 mm height (3D scaffold); a one-layer disk-shaped scaffold with a diameter of 8 mm and height of 0.4 mm (2D membrane) was also prepared. Next, the printed collagen was immersed and cross-linked with OPCs for 4 h. Later, the C + OPC scaffolds were freeze-dried and re-crosslinked for 1 h with three different OHA concentrations. Following this step, the scaffolds were freeze-dried again. Finally, four different compositions of the composite scaffolds were obtained: C + OPCs and C + OPCs + OHA (1, 5, and 10 mg/mL).

### 2.3. Characterization of Materials

#### 2.3.1. Fourier Transform Infra-Red (FTIR)

Functional groups and molecular interactions in the OHA were evaluated by FTIR. This experiment was performed using an FTIR spectrometer (FT720, JASCO, Easton, MD, USA) with wavelengths in the range of 400‒4000 cm^−1^ and a resolution of 8 cm^−1^.

#### 2.3.2. Scanning Electron Microscopy (SEM)

SEM (JEOL, JSM-7100F, Tokyo, Japan) was used to observe the surface morphology, amount of apatite, and cell adhesion on the composite scaffolds. All the specimens were sputter-coated with gold prior to observation.

#### 2.3.3. Mechanical Properties of the Scaffolds

The mechanical strength of the 2D membrane and 3D scaffolds, with a diameter of 8 mm and thicknesses of 0.4 and 1.2 mm, respectively, was measured using a compressive test (Deben MICROTEST, London, UK). After preconditioning, the samples were compressed at a rate of 0.01 mm/s.

#### 2.3.4. Rheological Properties of the Hydrogel

The viscosity and modulus of the collagen hydrogel were measured by a Modular Compact Rheometer (MCR 302, Anton Paar, Graz, Austria) using the measuring cone CP25-1 for 8.2 min at 25°C. The complex viscosity and modulus changes over shearing frequency were recorded with Start Rheoplus software (V3.62, Anton Paar GmbH, Graz, Austria).

### 2.4. In Vitro Experiments

#### 2.4.1. Degradation Rate

Degradation tests of the composite scaffolds with different OHA concentrations (0, 5, and 10 mg/mL) were conducted by immersing the scaffolds in PBS (pH 7.4) at 37 °C for different time intervals (14, 49, and 63 days). The degradation rate was calculated as follows:

(1) $$\mathrm{Mass}\;\mathrm{loss}=\left(W_{d}-{\;W}_{t}\right)/W_{d}$$

where W_d_ is the initial dry weight of the sample, and W_t_ is the weight of the sample after removing the solution (degraded samples).

#### 2.4.2. Bioactivity Evaluation

The bioactivity of the scaffolds was determined by the formation of an apatite-like phase. The samples were dipped into a two-times concentrated simulated body fluid at 37 °C for 7, 14, and 21 d. The surface morphology of the specimens with apatite was observed using SEM. The apatite crystal structure on the scaffolds was assessed using XRD (X’Pert3 Powder, PANalytical, Almelo, The Netherlands). Data were obtained in the 2θ range of 20–60° with a scanning rate of 10°/min and an interval of 0.02°.

#### 2.4.3. Cell Cytotoxicity and Cell Viability

The cytotoxicity of the scaffolds was assessed using the Cell Counting Kit-8 (CCK-8). It uses a tetrazolium salt, WST-8, which produces water-soluble WST-8 formazan. Since the orange-colored formazan does not require dissolution, no solubilizing process was required. Therefore, the formazan directly reflects the number of viable cells present in a sample. The more the cells proliferate, the darker the color. However, the greater the cell toxicity, the lighter the color. Absorbance was measured at 450 nm using a microplate reader (Sunrise remote F039300, Tecan, Männedorf, Switzerland).

Cell viability was assessed using an immunofluorescence assay. DAPI (diamidino-2-phenylindole) blue was used as a fluorescence dye to explore cell adhesion on the 2D membrane and 3D scaffolds. As an essential step, the specimens were UV-sterilized for 30 min and placed into 48-well plates containing 200 μL stem cell culture solution. The medium was replaced with fresh stem cell culture medium (1 mL), and rat bone marrow mesenchymal stem cells at a density of 5 × 10^4^ per well were seeded on the scaffolds for three days. Next, the culture solution was removed and the cells were washed with PBS. DAPI (500 μL) was added to each well at room temperature, and the plates were incubated for 15 min. Thereafter, the scaffolds were washed thrice with PBS. A fluorescence microscope (50i, Nikon, Tokyo, Japan) was used to observe cell attachment and volume changes at wavelengths ranging from 420 nm to 485 nm. To investigate cell adhesion, the cell-loaded scaffolds were prepared for SEM analysis. The cells on the scaffolds were then cured with 2.5% glutaraldehyde. Subsequently, the specimens were coated with gold and observed using SEM.

### 2.5. In Vivo Tests

SD rats (body weight: 75–95 g, 4-weeks-old) were used for in vivo experiments. The SD rats were acclimatized for at least one week before the experiment. The experiments were carried out at the Chang Gung Memorial Hospital according to guidelines for the care and use of animals. All experiments were approved by the Affiliated Institutional Animal Care and Use Committee (IACUC) under affidavit no. 2019,102,401. A diet of rat chow was provided ad libitum with a continuous supply of water. The animals were anesthetized by intraperitoneal injection of a 0.3 mL mixture of Zoletil 50 (Virbac, Carros, France) and Rompun 20 (Bayer, Leverkusen, Germany) at a ratio of 1:2. The scalps were shaved and the skins were sterilized using 70% ethanol. A sagittal incision was made on the pre-treated scalps. After subperiosteal undermining, approximately 6.0 mm diameter circular bone defects were made in the parietal bones using a dental trephine bur. The defects were adequately washed with saline to remove the bone debris and blood. The experiments consisted of three groups of the rats (n = 5): the control group, and the collagen + OPC and the collagen + OPC + OHA groups, in which the scaffolds were implanted on the defects. After implantation, the periosteum and scalps were closed using 4-0 Vicryl sutures. The rats were subcutaneously administered buprenorphine analgesic 0.02–0.05 mg/kg every 8–12 h for pain management. The healing progress and body weights of the rats were monitored. In the first and fourth weeks, the rats were euthanized by CO_2_ inhalation. The calvaria were cut and stained with toluidine blue under a Nikon Eclipse 50i upright microscope (Nikon, Tokyo, Japan) for histological analysis. Data were reviewed by a medical doctor.

## 3. Results and Discussion

### 3.1. Characterizations of Collagen/OPC/OHA Composite Scaffolds

**Figure 1:**
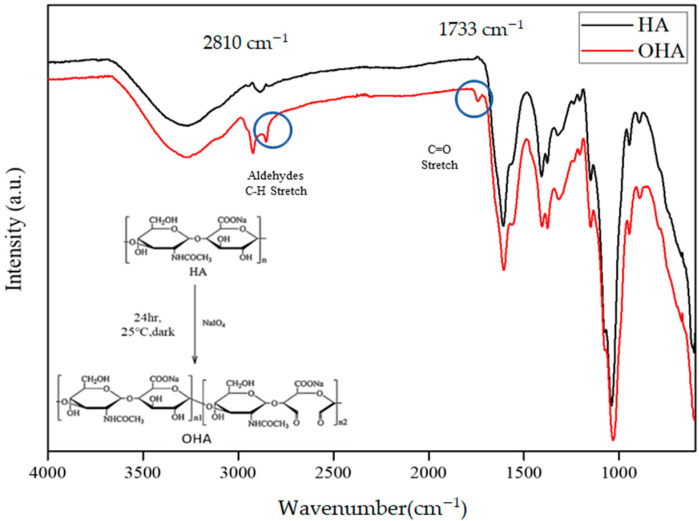
FTIR spectra of pure hyaluronic acid and oxidized hyaluronic acid (OHA).

FTIR analysis was performed to confirm the chemical bonding on OHA, and this was compared with pure HA (Figure 1). Sodium periodate was used to open the saccharide ring of the diols in HA to produce dialdehyde groups in OHA. The peak at 1733 cm^−1^ in the spectrum of OHA could be attributed to the stretching vibration absorption of the C=O aldehyde group, which was comparable to that of the original structure of the C=N stretching of the secondary amine group (CH_3_C(O)-NHCH), which was observed at 1650 cm^−1^_._ Further, the absorption peak at 2810 cm^−1^ corresponded to the CH stretch on the aldehyde group. The two aldehyde peaks that were observed in the FTIR spectra indicate the successful oxidation of hyaluronic acid in the scaffold system.

**Figure 2:**
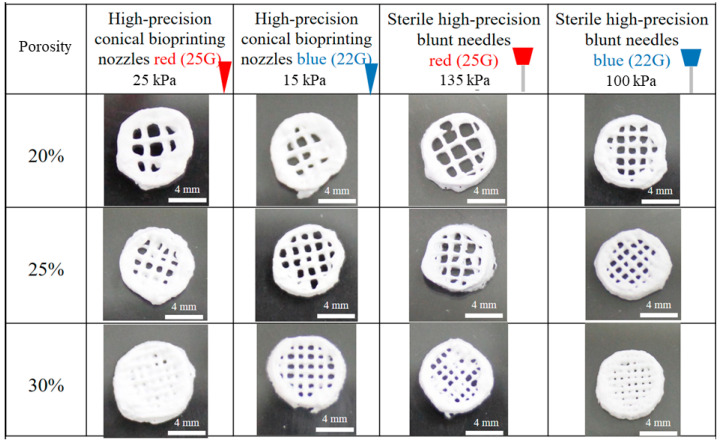
Optimization of 3D bioprinting parameters for obtaining porosity at 20%, 25%, and 30% using different needle densities (25G red plastic and metal, 22G blue plastic and metal) and different pressures (25, 15, 135, and 100 kPa).

C/OPC/OHA composite scaffolds were fabricated using a 3D bioprinter. Four types of needles, 25G red plastic, 22G blue plastic, 25G red metal, and 22G blue metal, were used to produce scaffolds with different porosities of 20%, 25%, and 30% (Figure 2). Pressures of 25, 15, 125, and 100 kPa were used for each needle. The 25G red and 22G blue needles had aperture sizes of 260 and 413 μm, respectively. The results showed that the 25% porosity scaffolds had a better porous structure than those with other porosities. In addition, the 22G blue needles only required four layers of printing to produce scaffolds, compared to the six layers required by 25G. Additionally, the 25G red needles showed a collapsed wall and required two more printed layers to reach a height of 1.2 mm. The pore size of the bone is approximately 20–1500 μm [35]. A pore size larger than 300 μm is beneficial for angiogenesis and bone generation [36]; however, pore sizes less than 300 μm can promote cartilage ossification [37]. Consequently, a 22G blue metal needle with 25% porosity was chosen as a printing parameter for creating the C/OPC/OHA scaffolds.

**Figure 3:**
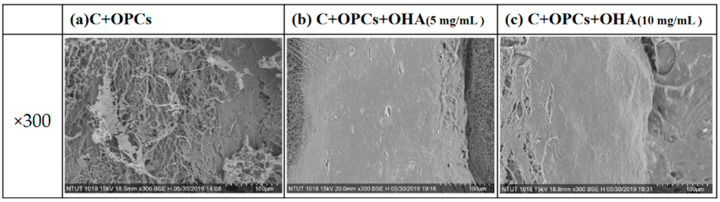
Surface morphologies of collagen + OPCs (**a**) and collagen + OPCs + OHA (5 mg/mL and 10 mg/mL) (**b**,**c**) observed by SEM (magnification 300×).

Figure 3 shows the surface morphology of three scaffold types: lyophilized C + OPC and C + OPC + OHA (5 and 10 mg/mL). The C + OPC scaffolds did not display cross-linked OHA; the collagen fibers were visible on the surface. However, both the C + OPC + OHA (5 and 10 mg/mL) scaffolds exhibited flatter and smoother surfaces as the concentration of OHA was increased.

**Figure 4:**
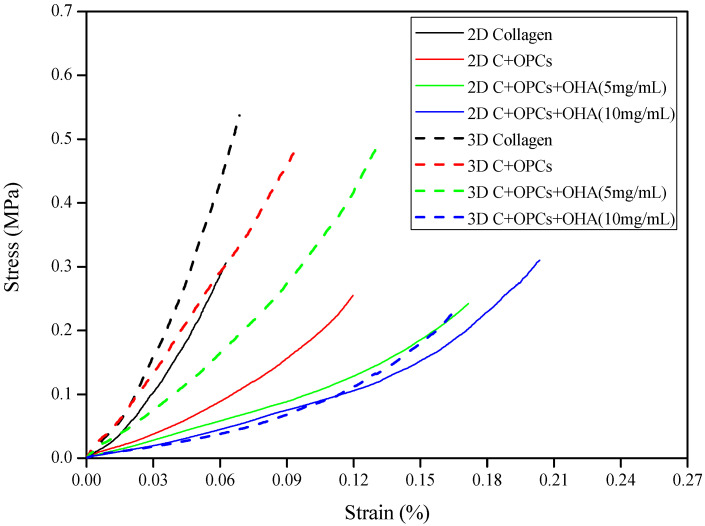
Compressive stress-strain curves of 2D membranes and 3D scaffolds with different concentrations of OHA.

The compressive stress-strain curves of the two-dimensional membranes and three-dimensional scaffolds are shown in Figure 4. All the curves are shown at logarithmic patterns and represent typical stress-strain curves for porous material [38]. Healthy cartilage has a compressive stress in the range of 0.1 to 20 MPa [15]. Our results demonstrated that both the 2D membranes and 3D scaffolds had a compressive stress between 0.25–0.55 MPa, which is still in the range of the compressive stress of normal cartilage. In addition, the 3D scaffolds were observed to have higher stress than the 2D membranes. This indicates that the 3D scaffolds had better mechanical properties; therefore, they could be used as an alternative for cartilage repair.

**Figure 5:**
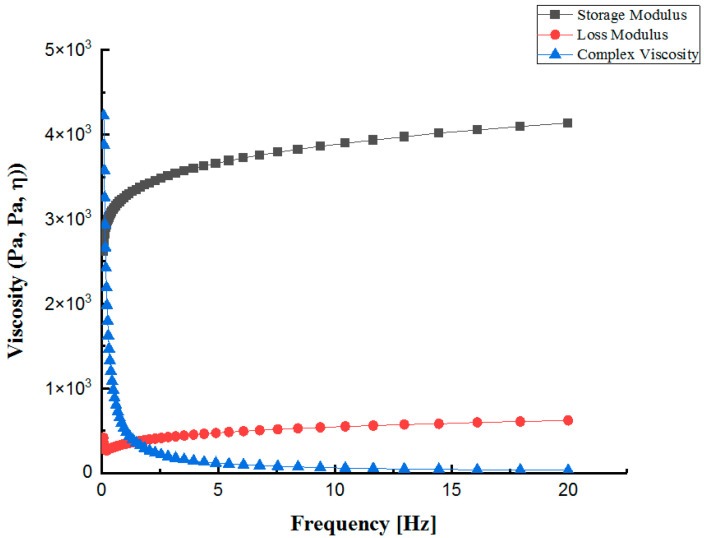
The rheological behavior of collagen hydrogel includes storage modulus (G’), loss modulus (G’’), and complex viscosity.

The rheological properties of collagen hydrogel were evaluated, and are shown in Figure 5. The storage modulus (G′) was always higher than the loss modulus (G′′). The minimum and maximum storage moduli of the hydrogel were approximately 2.6 kPa and 4.1 kPa, respectively. While the minimum loss modulus was about 270 Pa, it rose to its highest value at 620 Pa. Otherwise, the high complex viscosity [39] (around 4.2 kPa) of the bioink decreased according to higher shearing frequencies. These results proved that collagen hydrogel is suitable for extrusion 3D printing.

### 3.2. Degradation Behavior of Composite Scaffolds

**Figure 6:**
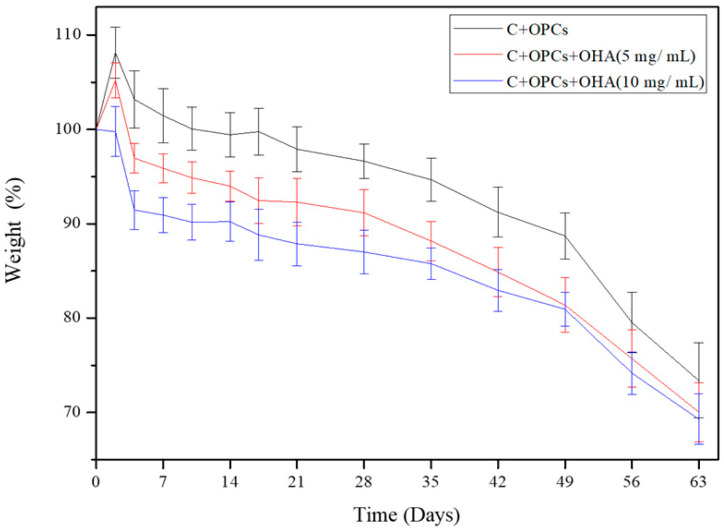
Degradation behavior of collagen-based scaffolds with different OHA concentrations in PBS solution at different time intervals.

The effects of OHA concentrations on the degradation behavior of the composite scaffolds were investigated. The correlation between mass loss and degradation time is shown in Figure 6. The degradation rate of the C + OPC scaffolds was not significant compared with that of the C + OPC + OHA scaffolds for concentrations of 5 mg/mL and 10 mg/mL for the first 14 days. The degradation rate values for both the scaffolds with OHA were 4.1% and 9.1%, respectively. After 49 days, the degradation rates increased to 26.6%, 30%, and 30.7% for 0, 5, and 10 mg/mL of OHA, respectively. An increase in the OHA concentration led to a slightly faster degradation rate. Although the degradation rate was higher, the cross-linking may not have caused the structure of the scaffolds to become unstable and easily degradable. Unfortunately, the degradation test could not be carried out after 63 days because the samples cracked and fractured due to their being freeze-dried and weighed several times.

### 3.3. Bioactivity and Cell Viability

**Figure 7:**
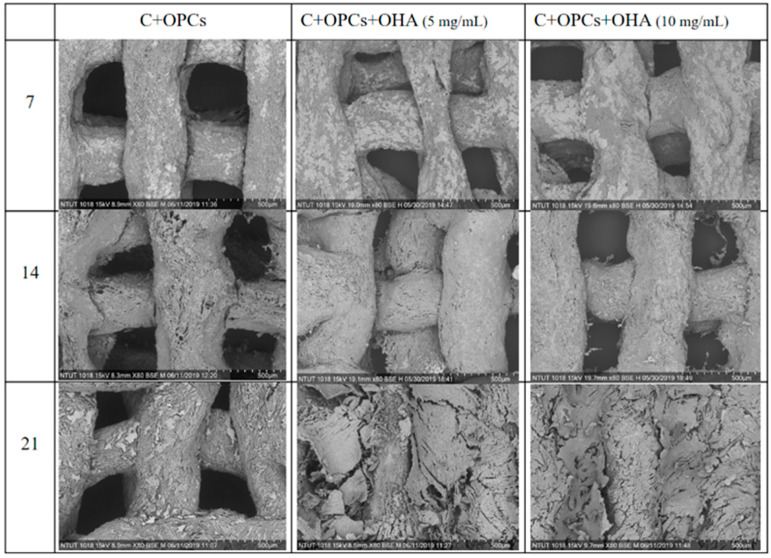
SEM images of collagen + OPC and collagen + OPC + OHA scaffolds after their immersion in simulated body fluid for 7, 14, and 21 days (magnification 80×).

The bioactivity of the scaffolds was determined by the presence of an apatite-like phase on the surface of the scaffolds. The samples were immersed in simulated body fluid for 7, 14, and 21 days. The samples were observed and characterized using SEM and XRD. As shown in Figure 7, the amount of apatite precipitated on the surface at day 7 was low. After 14 days, there was an improvement in the amount of apatite deposited, but it was not significant. However, the apatite precipitation was apparent in all the samples when the soaking time reached 21 days.

**Figure 8:**
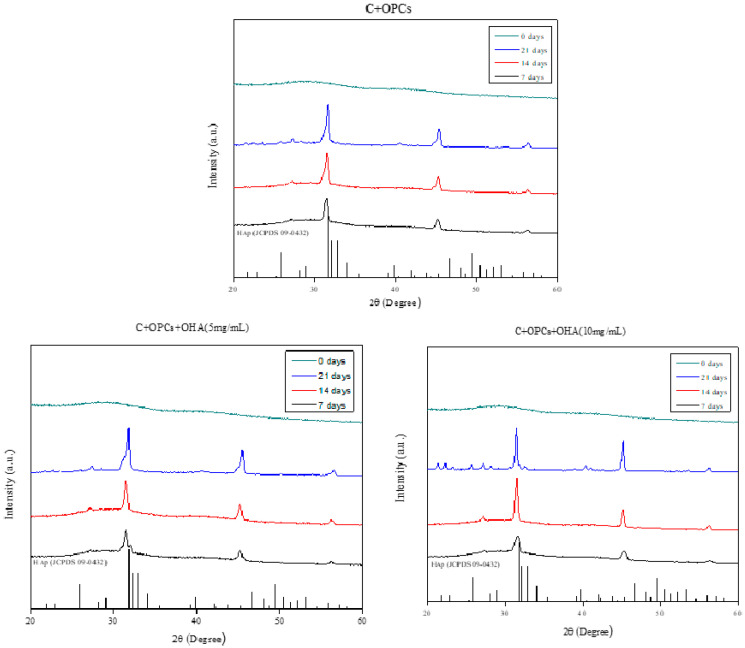
XRD analysis of scaffolds with different concentrations of OHA (0, 5, and 10 mg/mL) immersed in simulated body fluid for 0, 7, 14, and 21 days.

XRD was used to identify the apatite crystal structure in the scaffolds (Figure 8). The obvious characteristic peaks were observed at 2θ of 32°. According to JCPDS 09-0432, the characteristic peaks corresponded to apatite. The characteristic peaks significantly increased with increasing immersion time in simulated body fluid; these results agreed with the SEM results (Figure 3 and Figure 7). In addition, the apatite concentration was higher when the OHA concentration was increased. Taken together, the SEM and XRD results suggest that the C + OPC + OHA scaffolds have excellent bioactivity properties.

**Figure 9:**
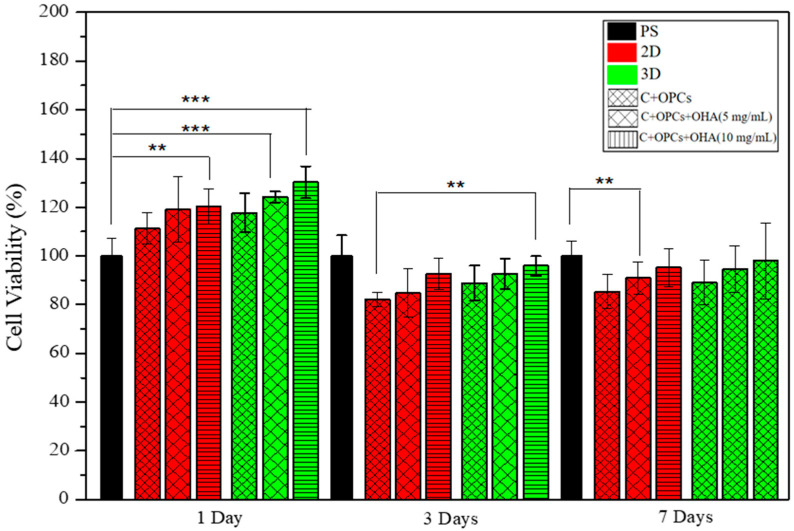
The cell counting kit-8 assay showed viable rat bone marrow mesenchymal stem cells after their being cultured on the 2D membrane and 3D scaffolds for 1, 3, and 7 days. (** and *** indicate *p* value < 0.01 and < 0.001).

Rat bone marrow mesenchymal stem cells (rBMSCs) were used for cell toxicity experiments using fluorescent staining (CCK-8 reagent). The cell viability results at different times (1, 3, and 7 days) are depicted in Figure 9. On day 1, the cell viability was higher than 100% because of the dissolution of some OPCs, resulting in a detection error. The cell viability observed for the 3D scaffolds was slightly higher than that for the 2D membrane at any point in time (days 3 and 7). The scaffolds with a C/OPC/OHA composition (10 mg/mL) displayed the highest cell viability (>90%), thus indicating that a higher OHA concentration results in a higher cell viability.

**Figure 10:**
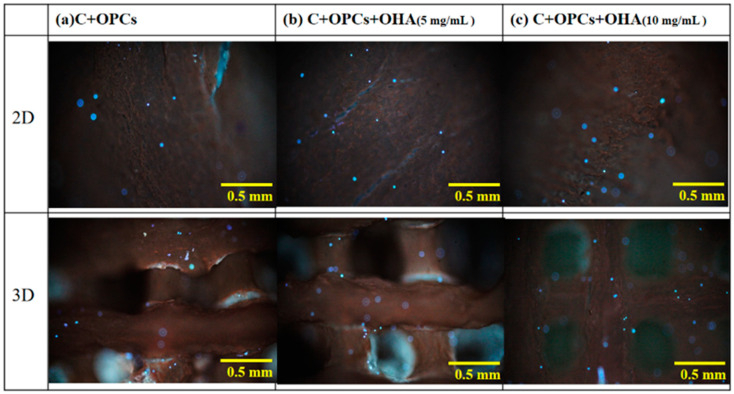
Fluorescence images of rat bone marrow mesenchymal stem cells on a 2D membrane and 3D scaffolds with different compositions and concentrations. Each fluorescence spot represents a stained stem cell nucleus.

**Figure 11:**
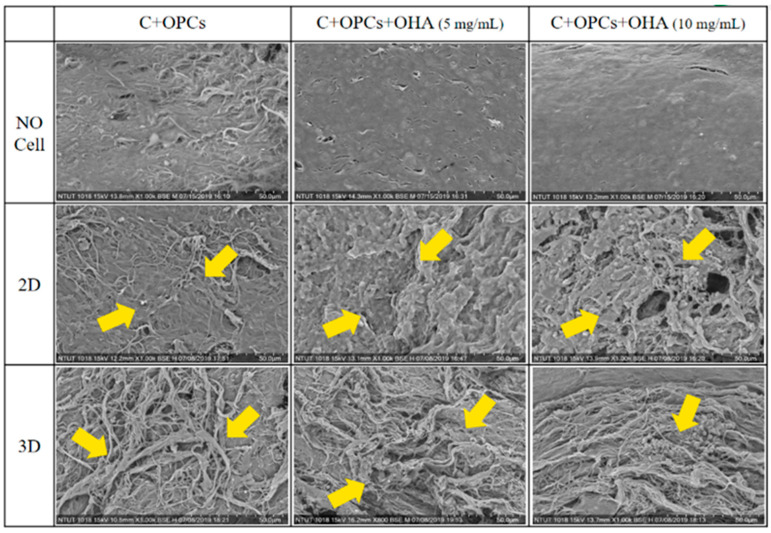
SEM of morphology of rBMSCs (yellow arrows) spread on the surface of a 2D membrane and 3D collagen scaffolds.

In addition, the rBMSCs were stained with DAPI blue to assess the attachment of the cells on the 2D membrane and 3D scaffolds. Fluorescence images of the samples are shown in Figure 10. These results were consistent with those of the cell toxicity results. A high number of cells were observed in the 3D scaffolds with the highest OHA content. These results were also supported by the SEM observations (Figure 11). More cells were attached to the 3D scaffolds with the composition of C + OPCs + OHA. The cells spread well on the scaffolds, and were fully extended and tightly attached to the surface, and more pseudopods protruded forward. However, on the surface of the 2D membrane C + OPCs, the cells were spread out without their showing obvious pseudopodia. These results suggest that because of the addition of OHA in the collagen scaffold system, the scaffolds showed low cell toxicity; OHA also provided a better cell proliferation environment to the cells.

### 3.4. In Vivo Evaluation

**Figure 12:**
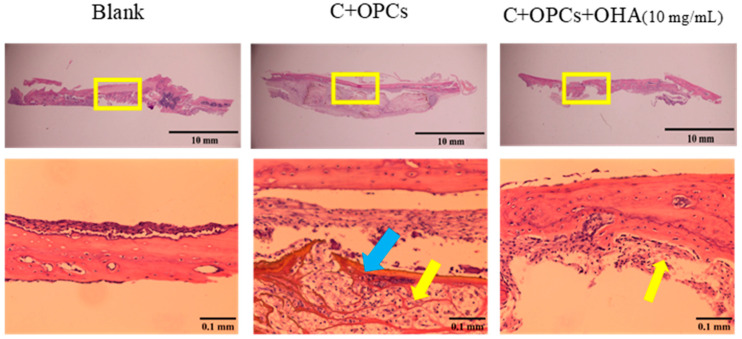
Histological images of control (Blank), collagen + OPC, and collagen + OPC + OHA scaffolds implanted on a skull defect captured after 1 week (upper panel). Enlarged images of the area within the yellow squares in the upper panels are shown in the lower panels (yellow arrows indicate the formation of new blood vessels, and the blue arrow indicates a scaffold).

**Figure 13:**
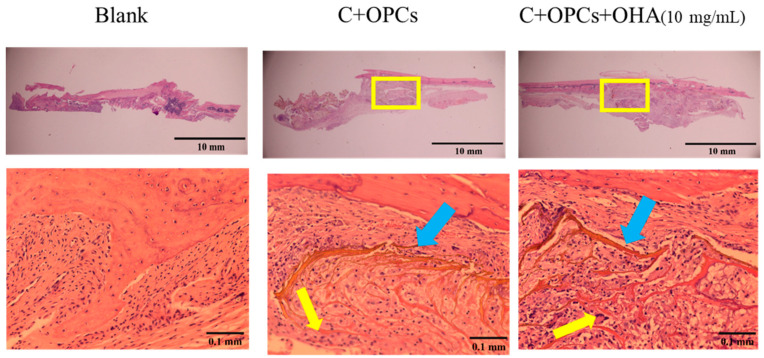
Histological images of control (Blank), collagen + OPC, and collagen + OPC + OHA scaffolds implanted on a skull defect captured after 4 weeks (upper panel). Enlarged images of areas within the yellow squares in the upper panels are shown in the lower panels (yellow arrows indicate the formation of new blood vessels, and the blue arrows indicate the scaffolds).

To assess the bone restoration capacity, C/OPC and C/OPC/OHA scaffolds were implanted into the bone defects in rat skulls at 1 week and 4 weeks. Histological images of bone restoration are shown in Figure 12 and Figure 13. The control group (Blank) demonstrated a normal inflammation reaction, and several macrophages accumulated around the defect sites. Angiogenesis and new bone restoration occurred in the C/OPC and C/OPC/OHA (10 mg/mL) scaffolds at 1 week and 4 weeks after implantation. It is well known that angiogenesis contributes to bone and tissue repair.

**Figure 14:**
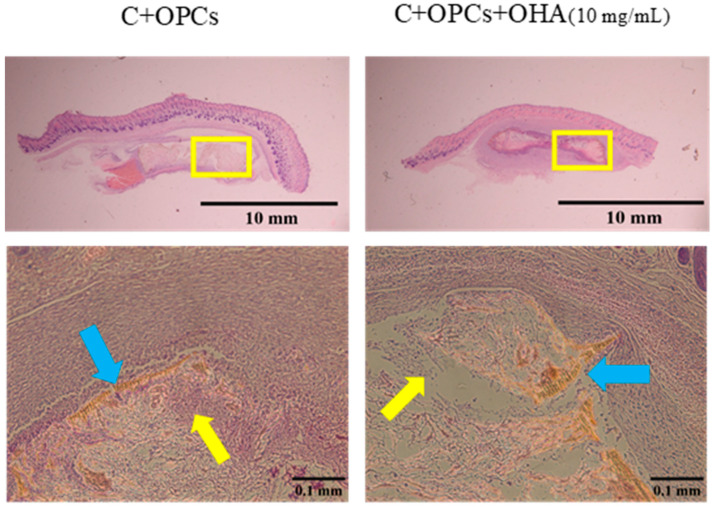
Histological images of collagen + OPC and collagen + OPC + OHA scaffolds implanted under the skull skin were captured after 1 week (upper panel). Enlarged images of areas within the yellow squares of the upper panels are shown in the lower panels (yellow arrows indicate new blood vessel formation, and the blue arrows indicate the scaffolds).

**Figure 15:**
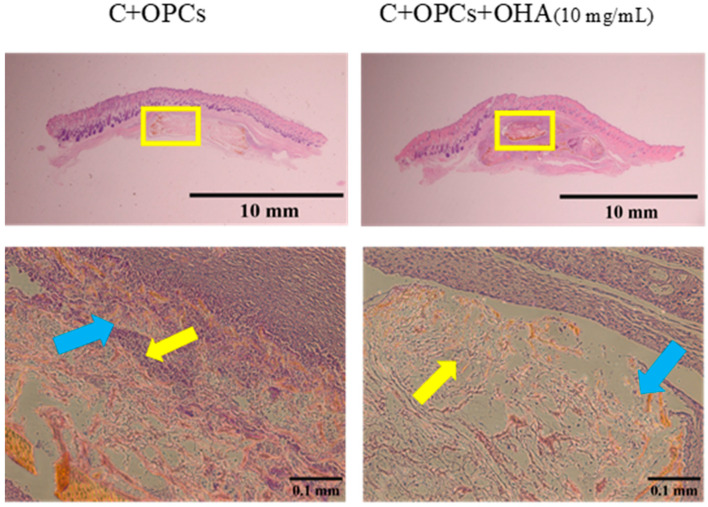
Histological images of control (Blank), collagen + OPC, and collagen + OPC + OHA scaffolds implanted on a skull defect captured after 4 weeks (upper panel). Enlarged images of areas within the yellow squares in the upper panels are shown in the lower panels (yellow arrows indicate the formation of new blood vessels, and blue arrows indicate the scaffolds).

The biocompatibility of the 3D scaffolds was investigated 1 week and 4 weeks after the scaffolds were implanted and placed under the skull skin (Figure 14 and Figure 15). New blood vessels were formed within 1 week of implantation, and the scaffolds retained their structure. After 4 weeks, some parts of the scaffolds were degraded; nevertheless, formation of tissue and blood vessels was still observed. Thus, collagen-based composite scaffolds with a composition of C + OPCs and C + OPCs + OHA (10 mg/mL) showed an excellent ability to support angiogenesis; therefore, they demonstrated a better ability to repair skull defects.

## 4. Conclusions

Collagen/proanthocyanidin/oxidized hyaluronic acid composite scaffolds were successfully prepared using a 3D bioprinter. The parameters chosen for printing were a pressure of 120 kPa (to maintain a porosity of 25%) and a 413 μm metal needle aperture to create 3D scaffolds with a diameter of 8 mm and a thickness of 1.2 mm. Characterization of the scaffolds, as well as in vitro and in vivo experiments, were performed. The composite scaffolds generated a high compressive stress, resulting in high mechanical properties. In addition, the scaffolds displayed excellent bioactivity due to the presence of apatite crystal structures on their surfaces. rBMSCs exhibited high cell viability but low toxicity, indicating that the attachment and spreading of these cells on the scaffolds were high. In vivo tests using SD rats were also performed. Scaffolds implanted in bone defects of rat skulls could trigger angiogenesis and the formation of new bone. Thus, composite scaffolds comprising collagen/proanthocyanidin/oxidized hyaluronic acid can be used as potential candidates to repair articular cartilage.

## Data Availability

Data can be available from the authors upon reasonable request.

## References

[1] Greenberg S.E., VanHouten J., Lakomkin N., Ehrenfeld J., Jahangir A.A., Boyce R.H., Obremksey W.T., Sethi M.K. (2016). Does Admission to Medicine or Orthopaedics Impact a Geriatric Hip Patient’s Hospital Length of Stay?. J. Orthop. Trauma.

[2] Curl W.W., Krome J., Gordon E., Rushing J., Smith B.P., Poehling G.G. (1997). Cartilage injuries: A review of 31,516 knee arthroscopies. Arthrosc. J. Arthrosc. Relat. Surg..

[3] Pathria M.N., Chung C.B., Resnick D.L. (2016). Acute and Stress-related Injuries of Bone and Cartilage: Pertinent Anatomy, Basic Biomechanics, and Imaging Perspective. Radiology.

[4] Hunziker E. (2002). Articular cartilage repair: Basic science and clinical progress. A review of the current status and prospects. Osteoarthr. Cartil..

[5] Wong C.-C., Chen C.-H., Chiu L.-H., Tsuang Y.-H., Bai M.-Y., Chung R.-J., Lin Y.-H., Hsieh F.-J., Chen Y.-T., Yan T.-L. (2018). Facilitating in vivo articular cartilage repair by tissue-engineered cartilage grafts produced from auricular chondrocytes. Am. J. Sports Med..

[6] Raynauld J.-P., Buckland-Wright C., Ward R., Choquette D., Haraoui B., Martel-Pelletier J., Uthman I., Khy V., Tremblay J.-L., Bertrand C. (2003). Safety and efficacy of long-term intraarticular steroid injections in osteoarthritis of the knee: A randomized, double-blind, placebo-controlled trial. Arthritis Rheum..

[7] Iannitti T., Lodi D., Palmieri B. (2011). Intra-articular injections for the treatment of osteoarthritis. Drugs R D.

[8] Kennedy M.I., Whitney K., Evans T., Laprade R.F. (2018). Platelet-Rich Plasma and Cartilage Repair. Curr. Rev. Musculoskelet. Med..

[9] Seo S.-S., Kim C.-W., Jung D.-W. (2011). Management of Focal Chondral Lesion in the Knee Joint. Knee Surg. Relat. Res..

[10] Mankin H.J. (1982). The response of articular cartilage to mechanical injury. J. Bone Jt. Surg..

[11] Everhart J., Campbell A.B., Abouljoud M.M., Kirven J.C., Flanigan D.C. (2019). Cost-efficacy of Knee Cartilage Defect Treatments in the United States. Am. J. Sports Med..

[12] Basad E., Wissing F.R., Fehrenbach P., Rickert M., Steinmeyer J., Ishaque B. (2015). Matrix-induced autologous chondrocyte implantation (MACI) in the knee: Clinical outcomes and challenges. Knee Surg. Sports Traumatol. Arthrosc..

[13] O’Brien F.J. (2011). Biomaterials & scaffolds for tissue engineering. Mater. Today.

[14] Kohane D.S., Langer R. (2008). Polymeric Biomaterials in Tissue Engineering. Pediatr. Res..

[15] Nguyen Q.T., Hwang Y., Chen A.C., Varghese S., Sah R.L. (2012). Cartilage-like mechanical properties of poly (ethylene glycol)-diacrylate hydrogels. Biomaterials.

[16] Yamazaki C.M., Kadoya Y., Hozumi K., Okano-Kosugi H., Asada S., Kitagawa K., Nomizu M., Koide T. (2010). A collagen-mimetic triple helical supramolecule that evokes integrin-dependent cell responses. Biomaterials.

[17] Chevallay B., Herbage D. (2000). Collagen-based biomaterials as 3D scaffold for cell cultures: Applications for tissue engineering and gene therapy. Med. Biol. Eng. Comput..

[18] Wolf K., Alexander S., Schacht V., Coussens L.M., von Andrian U.H., van Rheenen J., Deryugina E., Friedl P. (2009). Collagen-based cell migration models in vitro and in vivo. Semin. Cell Dev. Biol..

[19] Fraser J.R., Laurent T.C., Laurent U.B. (1997). Hyaluronan: Its nature, distribution, functions and turnover. J. Intern. Med..

[20] Raemdonck K., Martens T.F., Braeckmans K., Demeester J., De Smedt S. (2013). Polysaccharide-based nucleic acid nanoformulations. Adv. Drug Deliv. Rev..

[21] Collins M.N., Birkinshaw C. (2013). Hyaluronic acid based scaffolds for tissue engineering—A review. Carbohydr. Polym..

[22] Necas J., Bartosikova L., Brauner P., Kolar J. (2008). Hyaluronic acid (hyaluronan): A review. Vet. Med..

[23] Freeman B.D., Pinnau I. (1999). Polymer Membranes for Gas and Vapor Separation.

[24] Lin W., Liu Z., Kampf N., Klein J. (2020). The Role of Hyaluronic Acid in Cartilage Boundary Lubrication. Cells.

[25] Singh A., Corvelli M., Unterman S.A., Wepasnick K.A., McDonnell P.J., Elisseeff J.H. (2014). Enhanced lubrication on tissue and biomaterial surfaces through peptide-mediated binding of hyaluronic acid. Nat. Mater..

[26] Chen Z., Chan P., Ho K., Fung K.P., Wang J. (1996). Antioxidant activity of natural flavonoids is governed by number and location of their aromatic hydroxyl groups. Chem. Phys. Lipids.

[27] Bravo L. (1998). Polyphenols: Chemistry, dietary sources, metabolism, and nutritional significance. Nutr. Rev..

[28] Fine A.M. (2000). Oligomeric proanthocyanidin complexes: History, structure, and phytopharmaceutical applications. Altern. Med. Rev..

[29] Sugimoto E., Yamaguchi M. (2000). Stimulatory effect of daidzein in osteoblastic MC3T3-E1 cells. Biochem. Pharmacol..

[30] Kim S., Nimni M.E., Yang Z., Han B. (2005). Chitosan/gelatin-based films crosslinked by proanthocyanidin. J. Biomed. Mater. Res. Part B: Appl. Biomater..

[31] Han B., Jaurequi J., Tang B.W., Nimni M.E. (2003). Proanthocyanidin: A natural crosslinking reagent for stabilizing collagen matrices. J. Biomed. Mater. Res..

[32] Chen K.-Y., Shyu P.-C., Dong G.-C., Chen Y.-S., Kuo W.-W., Yao C.-H. (2009). Reconstruction of calvarial defect using a tricalcium phosphate-oligomeric proanthocyanidins cross-linked gelatin composite. Biomaterials.

[33] Chen K.-Y., Chung C.-M., Chen Y.-S., Bau D.-T., Yao C.-H. (2012). Rat bone marrow stromal cells-seeded porous gelatin/tricalcium phosphate/oligomeric proanthocyanidins composite scaffold for bone repair. J. Tissue Eng. Regen. Med..

[34] Ebenezar A.R., Srinivasan N., Manimaran V.S., Srinivasulu S., Mahalaxmi S. (2011). Application of a proanthocyanidin agent to improve the bond strength of root dentin treated with sodium hypochlorite. J. Conserv. Dent..

[35] Lee S.J., Lee I.W., Lee Y.M., Lee H.B., Khang G. (2004). Macroporous biodegradable natural/synthetic hybrid scaffolds as small intestine submucosa impregnated poly(d, l-lactide-co-glycolide) for tissue-engineered bone. J. Biomater. Sci. Polym. Ed..

[36] Kuboki Y., Jin Q., Takita H. (2001). Geometry of Carriers Controlling Phenotypic Expression in BMP-Induced Osteogenesis and Chondrogenesis. J. Bone Jt. Surg..

[37] Roosa S.M.M., Kemppainen J.M., Moffitt E.N., Krebsbach P.H., Hollister S.J. (2010). The pore size of polycaprolactone scaffolds has limited influence on bone regeneration in an in vivo model. J. Biomed. Mater. Res. Part A.

[38] Shima S., Oyane M. (1976). Plasticity theory for porous metals. Int. J. Mech. Sci..

[39] Shin Y.J., Shafranek R.T., Tsui J.H., Walcott J., Nelson A., Kim D.-H. (2021). 3D bioprinting of mechanically tuned bioinks derived from cardiac decellularized extracellular matrix. Acta Biomater..

